# Political instability and child anemia in Peru: a national interrupted time-series analysis, 2006–2024

**DOI:** 10.3389/fpubh.2026.1880585

**Published:** 2026-08-04

**Authors:** Víctor Juan Vera-Ponce, Jhosmer Ballena-Caicedo

**Affiliations:** Facultad de Medicina (FAMED), Universidad Nacional Toribio Rodríguez de Mendoza de Amazonas (UNTRM), Chachapoyas, Peru

**Keywords:** anemia, child, preschool, health policy, infant, interrupted time series analysis, Peru, political instability

## Abstract

**Background:**

Peru underwent marked political instability after the 2016 electoral cycle. Whether that period coincided with a change in the national trajectory of child anemia among children aged 6–35 months has not been evaluated using a long national time series.

**Methods:**

We conducted a secondary analysis of the Demographic and Family Health Survey (ENDES), 2006–2024 (123,600 children aged 6–35 months with valid hemoglobin). We estimated weighted annual prevalences of anemia (hemoglobin <11.0 g/dL) and mean hemoglobin. The primary analysis used segmented interrupted time-series regression with a flexible pre-trend (knot in 2011), Prais–Winsten AR(1) correction, and a prespecified cut-point in 2017. Sensitivity analyses included alternative cut-points (2016, 2018, 2020), exclusion of 2020–2021, an interrupted time-series model with mean hemoglobin as a continuous outcome, and composition-adjusted predicted prevalence.

**Results:**

Anemia decreased from 56.4% (2006) to 41.4% (2011), plateaued (~43%) between 2012 and 2016, fell to 36.5% in 2021, and rebounded to 43.1% in 2023. The slope in 2006–2010 was −3.48 percentage points per year; in 2011–2016 it was near zero (−0.09 pp./year). In 2017 we observed a favorable level change relative to the counterfactual (−4.4 pp), followed by a positive and statistically significant change in slope (*β*₃ = 3.83; 95% CI 1.80–5.87; *p* = 0.001), consistent with loss of momentum and a subsequent rebound. The interrupted time-series model of mean hemoglobin showed directionally consistent results (slope change −0.137 g/dL/year; *p* = 0.014).

**Conclusion:**

The post-2016 period coincided with an initial decline relative to the counterfactual, followed by an adverse slope and a recent rebound. This pattern is compatible with programmatic fragility in a context of political and institutional instability, but should not be interpreted as proof of a direct causal effect of political instability itself. Anemia-control policies require mechanisms that protect operational continuity from political turnover and other concurrent health-system shocks.

## 1 Introduction

Anemia in early childhood remains a public health priority because it is associated with increased susceptibility to infections, impaired growth and neurodevelopment, and later losses in human capital and productivity (1–4). Although international guidelines have recently updated hemoglobin definitions and interpretation, anemia continues to be concentrated in low- and middle-income countries and in settings characterized by territorial and social inequalities (2, 5).

In Peru, child anemia has remained highly prevalent and unevenly distributed. National evidence documents a higher burden among younger children, poorer households, rural areas, and Andean and Amazonian territories (6, 7). These patterns occur within a health system that has achieved important gains in maternal and child indicators, yet remains fragmented, with access gaps and heterogeneous service capacity across territories (4, 8, 9).

Over the past decade, the Peruvian state promoted multiple interventions to prevent and control anemia, including screening, iron supplementation, growth and development monitoring (CRED), dietary counselling, and multisectoral strategies (10, 11). These instruments included the 2017 national technical standard for the preventive and therapeutic management of anemia, the 2018 Multisectoral Plan against Anemia, and COVID-19-era technical adaptations to maintain anemia prevention and control services (12–15). However, policy implementation coexisted with high ministerial turnover, conflict between branches of government, institutional crises, and subsequently the health, social, and economic shock of the COVID-19 pandemic (8, 9, 16). In this context, it is plausible that programmatic gains that depend on operational continuity, local supervision, and regular supply chains became more fragile.

Previous studies in Peru have been useful for describing shorter-term trends, socioeconomic inequalities, and associations with specific interventions; however, they have not comprehensively evaluated whether the post-2016 period coincided with a change in the national trajectory of child anemia using a long, nationally representative series (6, 7, 10). Accordingly, our primary objective was to describe the annual evolution of child anemia in Peru between 2006 and 2024 and to assess whether the post-2016 period was associated with changes in the level and slope of the national series among children aged 6–35 months. Secondary objectives were to evaluate annual anemia severity, mean hemoglobin as a continuous outcome, and the robustness of findings through sensitivity analyses with alternative cut-points and a composition-adjusted predicted prevalence series.

## 2 Methods

### 2.1 Study design

We conducted an analytical observational study based on secondary analysis of repeated cross-sectional microdata from Peru’s Demographic and Family Health Survey (ENDES). The descriptive component used annual survey estimates, whereas the primary inferential component relied on a national annual time series analysed using interrupted time-series (ITS) models. The analytic unit for the time-series models was the calendar year. Additionally, this study was reported in accordance with the STROBE guidelines (17), and the completed checklist is provided in Supplementary Text S1.

### 2.2 Data source, setting, and study period

ENDES is a probabilistic, stratified, multistage, cluster survey with annual national representativeness and representativeness for key geographic domains in Peru. For this analysis, we combined rounds from 2006 to 2024. The original master file included records from 2005, but that year was not used in the final series because it contributed no observations that simultaneously met the age criteria, had valid hemoglobin, and had complete survey design information (18).

### 2.3 Analytic population, sample, and selection criteria

The target population comprised children aged 6–35 months surveyed in ENDES. We restricted the sample to this age group because of its programmatic relevance, historically higher anemia burden, and better comparability with recent Peruvian literature (7). Selection criteria were applied sequentially: (1) valid survey year between 2006 and 2024; (2) age 6–35 months; (3) availability of valid child hemoglobin after cleaning implausible codes and standardizing the measurement scale; and (4) availability of primary sampling unit, stratum, and a positive sampling weight. We did not impute missing data; analyses used complete cases for each final outcome.

### 2.4 Harmonization of variables and outcome definitions

Harmonization decisions were prespecified and implemented entirely via scripts. The survey year variable was derived from the original round-specific variables. Survey design variables were harmonized prioritizing cluster identifiers, strata, and positive sampling weights; when weights were stored in the DHS integer format, they were rescaled by dividing by 1,000,000.

The primary outcome was binary child anemia, defined as hemoglobin <11.0 g/dL. We retained this cut-off to preserve historical comparability with ENDES, national surveillance, and the Peruvian literature, while acknowledging ongoing methodological discussions following the 2024 WHO guideline (2, 5, 19). Across all survey rounds, the primary anemia outcome was constructed using the same analytical threshold to preserve comparability. Child hemoglobin was harmonized prioritizing child-specific variables already adjusted for altitude; annual traceability showed that >99% of observations used these variables in all years. Harmonization addressed changes in variable names, storage scale, and availability of child-specific altitude-adjusted hemoglobin variables. We did not identify any survey year in which a different anemia classification criterion had to be applied for the primary analysis. Implausible values after conversion were treated as missing.

Secondary outcomes were child anemia severity—no anemia (≥11.0 g/dL), mild (10.0–10.9), moderate (7.0–9.9), and severe (<7.0)—and the annual mean hemoglobin as a continuous variable, used in a supplementary ITS model to verify that findings did not depend exclusively on the binary cut-off.

### 2.5 Temporal exposure and contextual variables

The primary temporal exposure was defined at the annual level. The main cut-point was prespecified as 2017 to represent the beginning of the post-2016 political period rather than a discrete policy intervention occurring at a single point in time. This specification should therefore be interpreted as a parsimonious temporal contrast between the pre-2017 trajectory and the subsequent period characterized by greater executive instability, executive–legislative conflict, forced presidential transitions, ministerial turnover, social unrest, and deterioration of political and health governance. Annual chronology of presidents, type of transition, number of Ministers of Health, and mean days per minister were compiled for descriptive and contextual purposes. The exploratory instability index summarized the relative annual intensity of institutional disruption using the same chronology and governance markers, but because it was not a validated measurement instrument, it was used only to support descriptive contextualization. These indicators were not modelled as individual-level exposures, were not intended to identify the independent effect of any single political event, and did not replace the primary ITS cut-point.

### 2.6 Statistical analysis

All analyses were executed using scripts in Stata 17.0 and R 4.3.1. Prevalences, means, and confidence intervals incorporated the complex ENDES survey design (cluster, strata, and weights), with correction for single-unit strata. For each year we obtained the sample size, weighted anemia prevalence with 95% CI, and mean hemoglobin.

The primary analysis used segmented interrupted time-series regression applied to the national annual prevalence. Unlike the initially proposed simple linear specification, the final model incorporated a flexible pre-trend with a knot in 2011. This knot was selected because the observed pre-2017 series showed a clear empirical transition from a rapid decline during 2006–2010 to a high plateau during 2011–2016, making a single pre-2017 linear counterfactual implausible. The knot was used to improve representation of the pre-interruption trajectory and was not intended to identify a separate policy intervention in 2011. The specification included: continuous time since 2006, an additional time term starting in 2011, a post-2017 indicator, and time elapsed since 2017. Annual prevalence estimates were weighted by the inverse variance of their standard errors. Serial correlation was modelled using Prais–Winsten AR(1). Main estimands derived from the fitted model included: the slope in 2006–2010, the slope in 2011–2016, the immediate level change in 2017 relative to the counterfactual, the post-2017 slope, the slope-change coefficient (*β*₃), and the adjusted gap relative to the counterfactual in 2024.

Sensitivity analyses replicated the same framework using alternative cut-points in 2016, 2018, and 2020, as well as a model excluding 2020–2021. As complementary analyses, we ran an ITS model with mean hemoglobin as a continuous outcome and an annual series of composition-adjusted predicted prevalence based on an individual-level model with year indicators and child- and household-level covariates. Model adequacy for the primary ITS was assessed using the AR(1) parameter *ρ*, the Durbin–Watson statistic, and the Ljung–Box Q (4) test.

To assess whether the primary slope-change estimate was driven by individual annual observations, we conducted a leave-one-year-out influence analysis. The primary ITS model was re-estimated after sequentially excluding each calendar year from 2006 to 2024. For each refitted model, we extracted the post-2017 slope-change coefficient (*β*₃), 95% CI, *p*-value, absolute post-2017 slope, and change relative to the full primary model.

### 2.7 Ethical considerations

This study consisted of a secondary analysis of fully anonymized microdata derived from a population-based survey with authorized access. Because there was no direct contact with participants and no primary data collection, this research did not require review or approval by an institutional ethics committee (20). Nevertheless, all data-handling procedures were conducted in strict accordance with the ethical principles of the Declaration of Helsinki to safeguard confidentiality.

## 3 Results

### 3.1 Analytic sample

Of 250,699 initial child records, 248,556 corresponded to 2006–2024, 130,663 to children aged 6–35 months, 124,210 had valid hemoglobin, and 123,600 comprised the final analytic sample with complete survey design information (Supplementary Table S1; Supplementary Figure S3). These sequential exclusions reflect the target age range, availability of valid hemoglobin, and completeness of the survey design variables required for weighted national estimates.

### 3.2 Political and health-governance context

Table 1 summarizes the annual political and health-governance context over the analytic period. Between 2006 and 2016, presidential continuity predominated, with instability mainly limited to regular electoral transitions and 1–2 Ministers of Health per year. From 2017 onward, executive–legislative confrontation, forced presidential transitions, dissolution of Congress, interim governments, social unrest, and high ministerial turnover became more frequent. The number of Ministers of Health reached four in 2018 and five in 2020–2022, with mean ministerial tenures close to 81 days in the most unstable years. These indicators were used to contextualize the post-2016 period, not as individual-level exposures or as formal causal mediators.

**Table 1:** Political chronology and health-governance indicators in Peru, 2006–2024.

Year	President(s)	Type of transition	No. of presidents	No. of ministers of health	Mean days per minister	Instability index (0–10)
2006	Toledo → García	Regular election	2	2	183	0.0
2007	García	Stable	1	1	365	0.0
2008	García	Stable	1	1	366	0.0
2009	García	Stable	1	2	243	0.8
2010	García	Stable	1	1	365	0.0
2011	García → Humala	Regular election	2	2	183	0.0
2012	Humala	Stable	1	2	244	0.8
2013	Humala	Stable	1	2	243	0.8
2014	Humala	Stable	1	2	243	0.8
2015	Humala	Stable	1	1	365	0.0
2016	Humala → Kuczynski	Regular election	2	2	183	0.0
2017	Kuczynski	Executive–legislative confrontation	1	2	183	1.5
2018	Kuczynski → Vizcarra	Forced resignation	2	4	104	5.5
2019	Vizcarra	Dissolution of Congress	1	2	183	1.5
2020	Vizcarra → Merino → Sagasti	Vacancy (impeachment) + interim government + COVID-19	3	5	81	10.0
2021	Sagasti → Castillo	Regular election + high turnover	2	5	81	3.5
2022	Castillo → Boluarte	Vacancy (irregular ouster)	2	5	81	7.0
2023	Boluarte	Social unrest	1	3	146	2.5
2024	Boluarte	Governance instability	1	2	244	1.0

The table summarizes annual contextual indicators used for descriptive purposes. “Stable” refers to presidential continuity within the calendar year; ministerial turnover could occur even in stable years. The instability index was an exploratory, non-validated contextual indicator intended to summarize the relative intensity of political disruption in descriptive terms. It did not replace the prespecified 2017 temporal cut-point and was not modelled as an individual-level exposure or mediator.

### 3.3 National trend in child anemia

**Figure 1:**
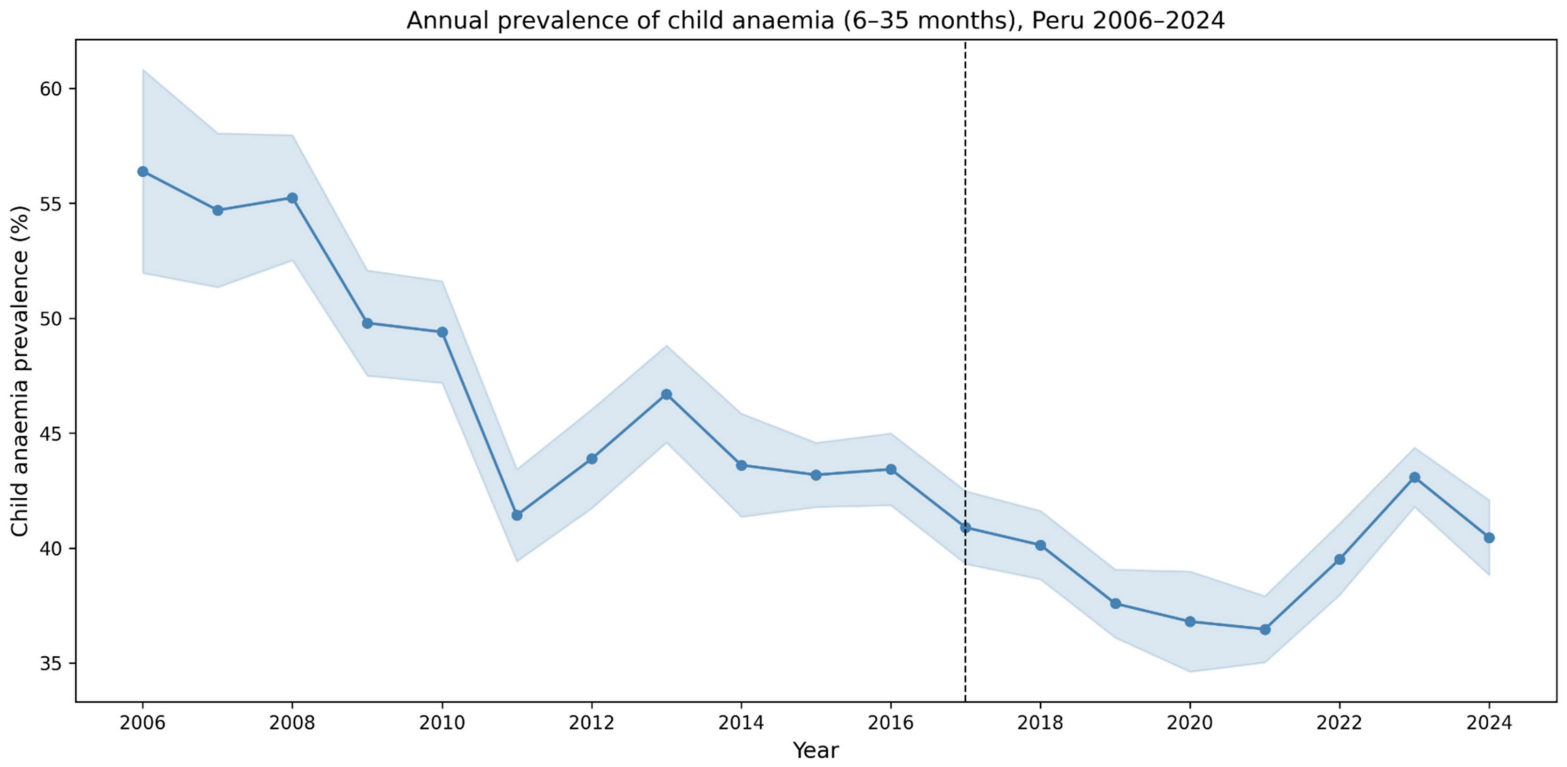
Weighted annual prevalence of child anemia among children aged 6–35 months, Peru 2006–2024. Shaded area: 95% CI. The vertical dashed line marks the primary cut-point (2017).

Figure 1 and Supplementary Table S2 show the weighted annual trajectory of child anemia. Prevalence decreased from 56.4% (95% CI 52.0–60.8) in 2006 to 41.4% (39.4–43.4) in 2011. Between 2012 and 2016, the series fluctuated in a high plateau (43.2–46.7%). In 2017, prevalence was 40.9% (39.3–42.5); it then declined to 36.5% (35.0–37.9) in 2021 and rebounded to 39.5% in 2022, 43.1% in 2023, and 40.5% in 2024. Mean hemoglobin followed an inverse pattern: it increased from 10.66 g/dL in 2006 to 11.11 g/dL in 2011, remained essentially stable between 2012 and 2016, and reached 11.32 g/dL in 2021 before declining to 11.05 g/dL in 2023 and 11.11 g/dL in 2024. The severity distribution also improved over the long term: the proportion without anemia increased from 43.6% in 2006 to 59.5% in 2024, while moderate or severe anemia fell from 26.4 to 13.7% (Supplementary Table S3).

### 3.4 Interrupted time-series analysis

**Figure 2:**
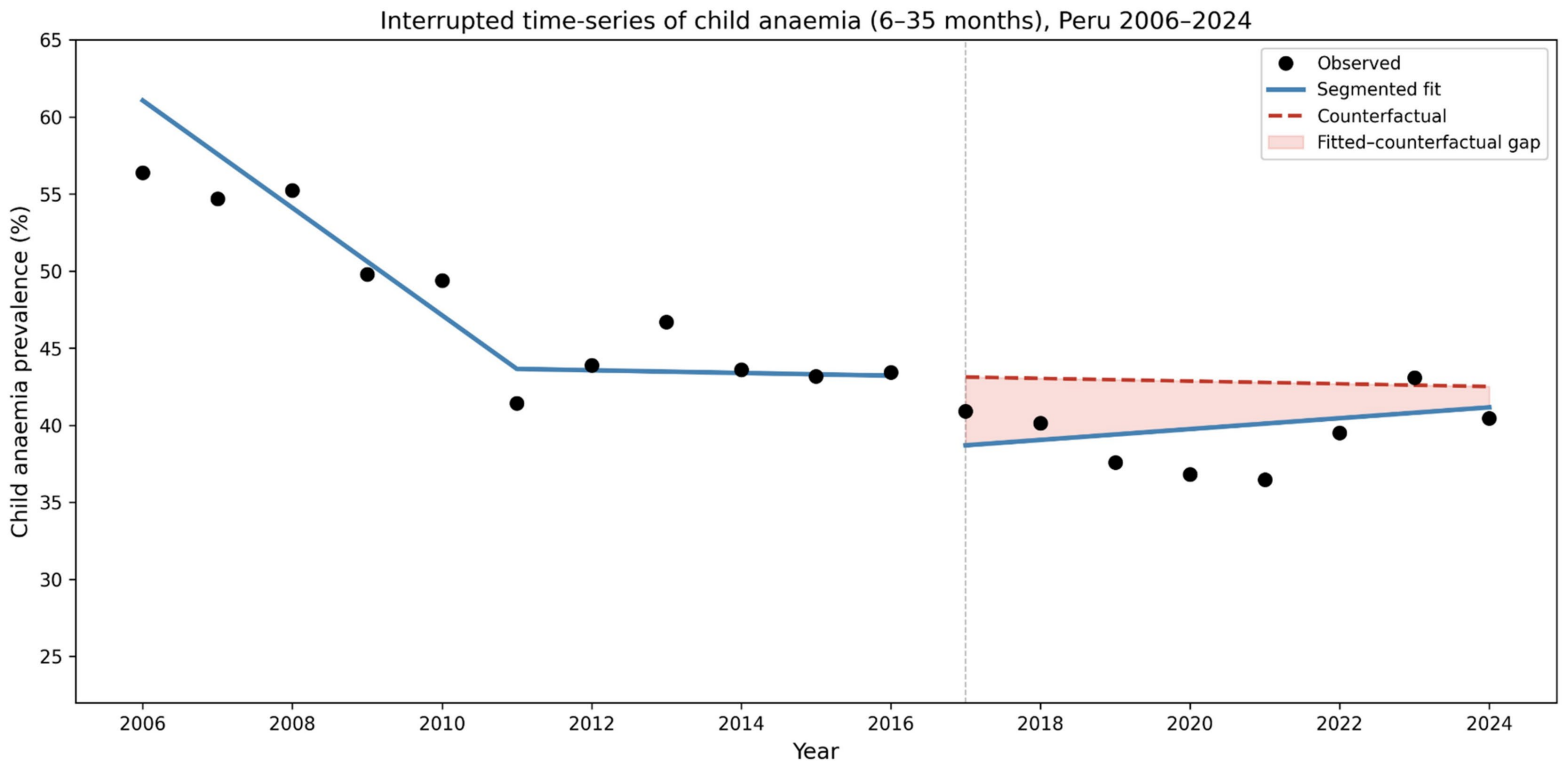
Interrupted time-series analysis of child anemia prevalence among children aged 6–35 months, Peru 2006–2024. Observed values (points), segmented fit (solid line), counterfactual since 2017 (dashed line), and the gap between both trajectories (shaded area) are shown.

Table 2 and Figure 2 summarize the primary model. The slope between 2006 and 2010 was markedly downward (−3.48 pp./year), whereas between 2011 and 2016 the series entered an almost flat plateau (−0.09 pp./year). Compared with the counterfactual projected from that plateau, we observed an immediate favorable level change of −4.43 percentage points in 2017. However, the slope-change coefficient was positive and statistically significant (*β*₃ = 3.83; 95% CI 1.80 to 5.87; *p* = 0.001), and the post-2017 slope was upward (+0.35 pp./year), consistent with loss of momentum from the prior decline and a recent rebound. In 2024, the model-adjusted prevalence remained 1.35 percentage points below the counterfactual, suggesting that the central finding was not an immediate worsening in 2017 but rather a less favorable subsequent trajectory. Model diagnostics are reported in Supplementary Table S4.

**Table 2:** Interrupted time-series results for child anemia prevalence: primary model and sensitivity analyses using alternative cut-points.

Model	Slope 2006–2010 (pp/year)	Slope 2011–year before cut-point (pp/year)	Level change at cut-point vs CF (pp)	Post-cut slope (pp/year)	β₃ (95% CI)	p (β₃)	Gap in 2024 vs CF (pp)
Cut-point 2016	−4.16	−0.18	−1.2	0.02	4.19 (1.27 to 7.11)	0.008	0.4
Cut-point 2017 [primary]	−3.48	−0.09	−4.4	0.35	3.83 (1.80 to 5.87)	0.001	−1.4
Cut-point 2018	−3.02	−0.33	−4.6	0.67	3.69 (2.20 to 5.18)	<0.001	1.4
Cut-point 2020	−2.42	−0.83	−2.8	1.95	4.37 (2.89 to 5.84)	<0.001	8.3

Models were estimated using segmented regression, a flexible pre-trend (knot in 2011), and Prais–Winsten AR(1) correction. CF = counterfactual. The sensitivity analysis excluding 2020–2021 is reported in the text (β₃ = 3.56; 95% CI 2.30 to 4.82; *p* < 0.001). In the primary model, *ρ* = 0.18, Durbin–Watson for transformed residuals = 1.60, and Ljung–Box Q(4) *p* = 0.689.

### 3.5 Additional and sensitivity analyses

The primary signal was robust across alternative cut-points (Supplementary Figure S1): *β*₃ was 4.19 (95% CI 1.27 to 7.11; *p* = 0.008) with a 2016 cut-point, 3.69 (2.20 to 5.18; *p* < 0.001) with a 2018 cut-point, and 4.37 (2.89 to 5.84; p < 0.001) with a 2020 cut-point. When excluding 2020–2021, the slope change remained positive and statistically significant (*β*₃ = 3.56; 95% CI 2.30 to 4.82; p < 0.001). These results reinforce the interpretation that the signal should be understood as a post-2016 phenomenon rather than a break attributable solely to a single year.

Leave-one-year-out diagnostics supported the robustness of the primary slope-change estimate. After excluding each calendar year one at a time, the post-2017 slope-change coefficient remained positive in all 19 refitted models and statistically significant in all cases. *β*₃ ranged from 2.88 to 4.60 percentage points/year. Excluding 2021, 2022, 2023, or 2024 did not reverse the sign of the coefficient, although the absolute post-2017 slope was most sensitive to exclusion of 2023. These findings indicate that the positive post-2017 slope-change coefficient was not driven by a single annual observation (Supplementary Table S6; Supplementary Figure S4).

The ITS model using mean hemoglobin as a continuous outcome was directionally concordant (Supplementary Table S5). The pre-2011 slope was upward (+0.125 g/dL/year; *p* = 0.018), the 2011–2016 phase remained essentially flat, there was no statistically significant level change in 2017 (−0.507 g/dL; *p* = 0.123), and the subsequent slope change was negative (*β*₃ = −0.137 g/dL/year; 95% CI − 0.241 to −0.033; *p* = 0.014). Overall, this concordance suggests that the signal does not depend exclusively on the dichotomous 11 g/dL threshold.

The sociodemographic composition–adjusted predicted prevalence series (Supplementary Figure S2) showed a pronounced decline through 2011, a plateau between 2011 and 2016, and a slightly lower level post-2017, with a mild decline through 2024. This pattern suggests that compositional changes in the sample are not sufficient on their own to explain the observed trajectory, although this sensitivity analysis should be interpreted as complementary rather than as a primary causal estimate.

## 4 Discussion

### 4.1 Main findings

This study shows that child anemia in Peru, assessed among children aged 6–35 months, had three distinguishable phases: an accelerated reduction between 2006 and 2010, a prolonged plateau between 2011 and 2016, and a post-2016 period characterized by an immediate decline relative to the counterfactual followed by a less favorable slope and a recent rebound. Substantively, the main signal should not be interpreted as a single shock in 2017, but as a trajectory compatible with fragility of prior progress and subsequent loss of momentum in a context of political and institutional instability.

Our interpretation also changed relative to the initially considered simple linear specification. A single pre-2017 line did not adequately represent the rapid early decline followed by a plateau. The final model with a knot in 2011 and Prais–Winsten AR(1) correction better describes the empirical history of the series and reduces concern about residual autocorrelation. Nonetheless, results should be read as evidence of a trajectory change compatible with the post-2016 period rather than as proof of a point-specific causal effect.

The directional concordance with mean hemoglobin strengthens interpretation. For both outcomes, we observed clear improvement in the early phase, stabilization in the intermediate phase, and a less favorable subsequent signal. The fact that alternative cut-points also yielded positive *β*₃ coefficients suggests a broader temporality of the phenomenon: rather than a break unique to 2017, it appears to be an accumulative process of the post-2016 period.

The leave-one-year-out diagnostics further support this interpretation. The post-2017 slope-change coefficient remained positive and statistically significant after sequential exclusion of every individual survey year, including the recent rebound years. This does not eliminate uncertainty inherent to annual time-series modelling, but it reduces the likelihood that the primary slope-change estimate was driven by a single annual observation.

### 4.2 Comparison with other studies

Our findings align with recent Peruvian literature. Mougenot and Herrera-Añazco described a decrease in anemia among children aged 6–35 months between 2014 and 2019, consistent with the downward portion of our series before the most recent rebound (21). The contribution of this study is to extend observation through 2024 and show that this favorable trajectory was not sustained with the same intensity when the time window is expanded.

National evidence on inequalities is also consistent with this interpretive framework. Previous studies have shown a higher anemia burden in rural areas, Andean and Amazonian territories, and more socioeconomically disadvantaged groups (6, 7). Although our primary analysis was national, this background suggests that the loss of momentum in anemia control is unlikely to be distributed homogeneously across territories.

Our results are also compatible with literature on implementation capacity. Berky and colleagues observed that exposure to preventive interventions may be associated with a lower probability of anemia, but highlighted monitoring limitations for attributing changes to specific components (10). Complementarily, Castro-Mayta showed that mere coverage of growth and development monitoring does not guarantee a sustained population-level reduction in anemia (11). Our results are consistent with that reading: operational continuity, implementation quality, and territorial coordination matter as much as the formal existence of programs.

Finally, this interpretation is consistent with evidence on the fragility of the Peruvian health system in crisis contexts. Villar and colleagues’ analysis of the pandemic and the OECD review describe a system marked by fragmentation, inequality, and variable capacity to sustain complex responses (8, 9, 16). Our study does not demonstrate a direct causal relationship between each political event and child anemia, but it is consistent with the hypothesis that reducing anemia becomes more difficult when the institutional environment loses stability, coordination, and managerial continuity.

A plausible interpretation is that the post-2016 signal reflects implementation fragility rather than the formal disappearance of anemia-control policies. During this period, Peru maintained and reformulated anemia-related policy instruments, including national technical guidance for preventive and therapeutic management, multisectoral planning, screening, iron supplementation, growth and development monitoring, dietary counselling, and territorial coordination (12–15). Therefore, the relevant mechanism is unlikely to be the mere presence or absence of policy documents. Rather, anemia control depends on repeated operational processes: timely hemoglobin screening, uninterrupted availability of iron and multiple micronutrients, adequate counselling, follow-up of treated children, local supervision, and coordination between health services, social programs, and subnational governments. Political instability may weaken these processes through managerial turnover, shifting priorities, delays in procurement or budget execution, reduced supervision, and weaker intersectoral coordination. However, because ENDES does not directly measure these implementation pathways, this mechanism should be interpreted as a plausible explanation rather than as a directly estimated mediator.

### 4.3 Public health implications

The first implication concerns governance. Anemia-control policies should not depend on short political cycles or the tenure of specific authorities. If the post-2016 period coincided with loss of momentum and a recent rebound, then programmatic continuity should be an explicit public policy objective.

The second implication is operational. In child anemia, it is not enough to expand nominal coverage; what matters is how interventions are delivered, how they are supervised, and whether critical inputs arrive on time. Performance metrics should include continuity of treatment, timeliness of follow-up, quality of dietary counselling, and stability of iron and multiple micronutrient supply.

The third implication is analytical. Annual surveillance should combine dichotomous and continuous outcomes, document hemoglobin traceability, and maintain reproducible trend and equity analyses that can detect early loss of progress. The methodological debate prompted by the WHO update reinforces the need to carefully evaluate any definitional change before translating it without nuance into national surveillance (2, 5, 19, 22).

### 4.4 Limitations

This study has several limitations. First, it is based on repeated cross-sectional surveys rather than longitudinal follow-up of the same children and therefore does not support individual-level causal inference. Second, the exposure of interest—the post-2016 period—is an ecological temporal construct and does not directly capture intermediate mechanisms related to implementation, financing, or service quality. Third, although multiple sensitivity analyses were conducted, concurrent time-varying factors such as the pandemic, food inflation, poverty, or other economic shocks could still influence the series. Fourth, sensitivity analyses with alternative cut-points show limited temporal specificity; thus, results should be interpreted as a signal of the post-2016 period rather than evidence of a break unique to 2017. Fifth, the primary analysis was restricted to children aged 6–35 months, which improves programmatic coherence but limits automatic extrapolation to ages 36–59 months. Sixth, although the study covers nearly two decades, the ITS models were estimated using annual observations, which limited statistical power, model flexibility, and the ability to evaluate short-term fluctuations within each year. For this reason, the segmented models were specified parsimoniously and the results were triangulated with sensitivity and leave-one-year-out analyses rather than interpreted as high-resolution causal estimates. Seventh, although hemoglobin variables were harmonized consistently across ENDES rounds and the same anemia threshold was applied throughout the study period, unmeasured changes in fieldwork procedures, calibration, personnel training, or survey operations could have contributed to small temporal differences.

## 5 Conclusion

Overall, results suggest that Peru moved from an accelerated reduction in child anemia to a prolonged plateau and subsequently to a period with an immediate decline but an adverse subsequent slope and recent rebound. This trajectory coincided with high political and institutional instability, but the study design does not allow attribution of the observed changes to political instability alone. Programmatic priorities should not be limited to sustaining nominal coverage, but should also safeguard institutional continuity, managerial stability, and execution capacity for essential interventions targeting early childhood.

## Data Availability

Publicly available datasets were analyzed in this study. The ENDES microdata used in the study are publicly available through the INEI microdata repository at: https://proyectos.inei.gob.pe/microdatos/.
