# Supplementary material for: Political instability and child anemia in Peru: a national interrupted time-series analysis, 2006–2024
Source: Front Public Health. 2026 Aug 4;14:1880585. doi: 10.3389/fpubh.2026.1880585 (PMC13481375; doi:10.3389/fpubh.2026.1880585)
Supplement: Supplementary file 1 [file Data_Sheet_1.docx]

***Supplementary Material***

**Table S1.** Flow of selection of the analytic sample.

**Table S2.** Weighted annual prevalence of child anemia and mean hemoglobin, 2006–2024.

**Table S3.** Annual distribution of child anemia severity, 2006–2024.

**Table S4.** Diagnostics for the main ITS model.

**Table S5.** ITS with child mean hemoglobin as a continuous outcome.

**Table S6.** Leave-one-out estimates.

**Figure S1.** Sensitivity analysis: slope change (β₃) using alternative cut-points.

**Figure S2.** Composition-adjusted predicted prevalence.

**Figure S3.** Flow diagram of analytic sample selection.

**Figure S4.** Leave-one-out estimates of the post-2017 trend-change coefficient (β₃).

**Supplementary Text 1.** STROBE checklist (observational studies).

# Supplementary Material

## Supplementary Text 1. STROBE Checklist

| **No.** | **Recommendation** | **Location in the manuscript** |
| --- | --- | --- |
| **TITLE AND ABSTRACT** | | |
| **1** | (a) Indicate the study design in the title or the abstract using a commonly used term | Title: 'interrupted time-series analysis'; Abstract (Summary): 'segmented interrupted time-series regression' |
|  | (b) Provide in the abstract an informative and balanced summary of what was done and what was found | Summary: includes background, methods (n=123,600; Prais–Winsten AR(1)), key findings (β₃=3·83; p=0·001), and interpretation |
| **INTRODUCTION** | | |
| **2** | **Background/rationale:** Explain the scientific background and rationale for the investigation being reported | Introduction, paragraphs 1–3: burden of child anaemia, Peruvian context (territorial and social inequalities), interventions coexisting with political instability and COVID-19 |
| **3** | **Objectives:** State specific objectives, including any prespecified hypotheses | Introduction, final paragraph: primary objective (describe trajectory and assess post-2016 changes in level and slope) and secondary objectives (severity, mean haemoglobin, sensitivity analyses) |
| **METHODS** | | |
| **4** | **Study design:** Present key elements of study design early in the paper | Methods, 'Study design': analytical observational study, secondary analysis of repeated cross-sectional ENDES microdata, interrupted time-series design |
| **5** | **Setting:** Describe the setting, locations, and relevant dates, including periods of recruitment, exposure, follow-up, and data collection | Methods, 'Data source, setting, and study period': ENDES 2006–2024, probabilistic stratified multistage cluster survey, annual national representativeness; 2005 excluded with justification |
| **6** | **Participants:** (a) Give the eligibility criteria, and the sources and methods of selection of participants | Methods, 'Analytic population': children 6–35 months; sequential selection criteria (valid year, age, haemoglobin, survey design); no imputation |
| **7** | **Variables:** Clearly define all outcomes, exposures, predictors, potential confounders, and effect modifiers | Methods, 'Harmonization of variables': primary outcome (anaemia <11.0 g/dL), secondary outcomes (severity, mean Hb); 'Temporal exposure': 2017 defined as a period-level contrast, political chronology and ministerial turnover described as contextual indicators, exploratory index not modelled as an exposure or mediator |
| **8** | **Data sources/measurement:** For each variable of interest, give sources of data and details of methods of assessment (measurement) | Methods, 'Harmonization': haemoglobin prioritizing altitude-adjusted variables, traceability showing >99% from these variables, use of the same analytical threshold (<11.0 g/dL) across all rounds, and acknowledgement that subtle fieldwork or protocol changes cannot be fully excluded |
| **9** | **Bias:** Describe any efforts to address potential sources of bias | Methods: complex survey design (cluster, strata, weights); flexible pre-trend to avoid counterfactual inflation; AR(1) correction for autocorrelation; composition-adjusted sensitivity; exclusion of 2020–2021; leave-one-year-out diagnostics |
| **10** | **Study size:** Explain how the study size was arrived at | Methods, 'Analytic population' and Results, 'Analytic sample': 250,699 initial records → 123,600 analytic sample after sequential exclusions; flow diagram in Supplementary Figure S3 |
| **11** | **Quantitative variables:** Explain how quantitative variables were handled in the analyses | Methods, 'Harmonization': haemoglobin conversion (tenths to g/dL), altitude adjustment formula, implausible value exclusion; 'Statistical analysis': ITS terms (time, knot, post, time×post) |
| **12** | **Statistical methods:** (a) Describe all statistical methods, including those used to control for confounding | Methods, 'Statistical analysis': Prais–Winsten AR(1), inverse-variance weighting of annual prevalence estimates, flexible pre-trend (knot 2011), derived estimands, Taylor linearization for survey estimates; sensitivity and influence analyses |
|  | (b) Describe any methods used to examine subgroups and interactions | No subgroup or interaction analyses were specified for the primary ITS. Sensitivity analyses evaluated alternative cut-points (2016/2018/2020), exclusion of 2020–2021, continuous Hb ITS, composition-adjusted prevalence, and leave-one-year-out diagnostics. |
|  | (c) Explain how missing data were addressed | Methods, 'Analytic population': complete-case analysis; no imputation; flow diagram documenting exclusions at each stage |
|  | (d) If applicable, describe analytical methods taking account of sampling strategy | Methods, 'Statistical analysis': all prevalences and means incorporate complex ENDES survey design (cluster, strata, weights) with single-unit strata correction |
|  | (e) Describe any sensitivity analyses | Methods, 'Statistical analysis': alternative cut-points (2016, 2018, 2020), exclusion of 2020–2021, continuous Hb ITS, composition-adjusted predicted prevalence, and leave-one-year-out influence diagnostics |
| **RESULTS** | | |
| **13** | (a) Report numbers of individuals at each stage of study and reasons for non-participation | Results, 'Analytic sample': 250,699 → 248,556 → 130,663 → 124,210 → 123,600; Supplementary Figure S3 (flow diagram) |
|  | (b) Give reasons for non-participation at each stage | Results and Supplementary Figure S3: exclusions for year, age, invalid haemoglobin, and missing survey design variables |
|  | (c) Consider use of a flow diagram | Supplementary Figure S3 |
| **14** | (a) Give characteristics of study participants and information on exposures and potential confounders | Results, 'National trend': annual sample sizes and prevalences; Table 1: political chronology with presidents, ministers, transitions, and contextual instability index; Supplementary tables: annual outcome series and severity distribution |
|  | (b) Indicate number of participants with missing data for each variable of interest | Results, 'Analytic sample': sequential exclusion counts; Methods, 'Harmonization': haemoglobin source prioritization, threshold consistency, and measurement limitations |
| **15** | **Outcome data:** Report numbers of outcome events or summary measures | Results, 'National trend': annual weighted prevalences with 95% CI (2006: 56·4%; 2011: 41·4%; 2021: 36·5%; 2023: 43·1%; 2024: 40·5%); mean haemoglobin by year; severity distribution |
| **16** | (a) Give unadjusted estimates and, if applicable, confounder-adjusted estimates and their precision | Results, 'ITS analysis' and Table 2: derived estimands (slopes, level shift, β₃ with 95% CI and p-values) for primary and sensitivity models; composition-adjusted series as sensitivity |
|  | (b) Report category boundaries when continuous variables were categorized | Methods, 'Harmonization': anaemia <11·0 g/dL; severity: mild (10·0–10·9), moderate (7·0–9·9), severe (<7·0) |
| **17** | **Other analyses:** Report other analyses done—e.g., analyses of subgroups and interactions, and sensitivity analyses | Results, 'Additional and sensitivity analyses': alternative cut-points (Table 2), exclusion of 2020–2021, continuous Hb ITS, composition-adjusted prevalence, and leave-one-year-out diagnostics (Supplementary Table S6; Supplementary Figure S4) |
| **DISCUSSION** | | |
| **18** | **Key results:** Summarise key results with reference to study objectives | Discussion, 'Main findings': three phases (accelerated reduction, plateau, post-2016 decline with adverse slope and rebound); signal framed as compatible with programmatic fragility rather than a single shock or direct causal proof |
| **19** | **Limitations:** Discuss limitations of the study, taking into account sources of potential bias or imprecision | Discussion, 'Limitations': repeated cross-sections, ecological exposure, concurrent shocks, limited temporal specificity of alternative cut-points, age restriction to 6–35 months, limited degrees of freedom from annual observations, and possible unmeasured fieldwork/protocol changes |
| **20** | **Interpretation:** Give a cautious overall interpretation of results considering objectives, limitations, multiplicity of analyses, results from similar studies, and other relevant evidence | Discussion, paragraphs 2–6: model choice rationale, concordance with Hb, leave-one-year-out diagnostics, comparison with Mougenot, Al-Kassab-Córdova, Berky, Castro-Mayta, Villar, OECD, and policy-implementation mechanisms; causal language explicitly tempered |
| **21** | **Generalisability:** Discuss the generalisability (external validity) of the study results | Discussion, 'Comparison with other studies' and 'Public health implications': findings consistent with Peruvian literature; implications framed for Latin American governance contexts; age restriction noted as a limitation for extrapolation |
| **OTHER INFORMATION** | | |
| **22** | **Funding:** Give the source of funding and the role of the funders for the present study and, if applicable, for the original study on which the present article is based | Acknowledgments and 'Role of the funding source': Vice-Rectory for Research, UNTRM; funder had no role in design, analysis, interpretation, writing, or decision to submit |

## Table S1. Flow of selection of the analytic sample

| **Analytic step** | **n** |
| --- | --- |
| Initial pool of child records | 250,699 |
| Records from 2006–2024 | 248,556 |
| Children aged 6–35 months | 130,663 |
| Valid hemoglobin | 124,210 |
| Complete survey design information | 123,600 |

## Table S2. Weighted annual prevalence of child anemia and mean hemoglobin, 2006–2024

| **Year** | **n** | **Anemia prevalence, % (95% CI)** | **Mean hemoglobin, g/dL (95% CI)** |
| --- | --- | --- | --- |
| 2006 | 863 | 56.4 (52.0–60.8) | 10.66 (10.54–10.78) |
| 2007 | 1,731 | 54.7 (51.3–58.0) | 10.71 (10.60–10.81) |
| 2008 | 3,324 | 55.2 (52.5–58.0) | 10.67 (10.60–10.75) |
| 2009 | 3,853 | 49.8 (47.5–52.1) | 10.86 (10.80–10.93) |
| 2010 | 3,902 | 49.4 (47.2–51.6) | 10.87 (10.80–10.93) |
| 2011 | 3,960 | 41.4 (39.4–43.4) | 11.11 (11.06–11.17) |
| 2012 | 4,184 | 43.9 (41.7–46.0) | 11.05 (10.99–11.10) |
| 2013 | 3,861 | 46.7 (44.6–48.8) | 10.99 (10.94–11.05) |
| 2014 | 3,803 | 43.6 (41.4–45.9) | 11.05 (10.99–11.11) |
| 2015 | 10,669 | 43.2 (41.8–44.6) | 11.06 (11.02–11.10) |
| 2016 | 9,452 | 43.4 (41.9–45.0) | 11.04 (11.00–11.08) |
| 2017 | 10,094 | 40.9 (39.3–42.5) | 11.11 (11.07–11.15) |
| 2018 | 9,862 | 40.1 (38.6–41.6) | 11.13 (11.10–11.17) |
| 2019 | 9,695 | 37.6 (36.1–39.1) | 11.20 (11.17–11.24) |
| 2020 | 5,646 | 36.8 (34.6–39.0) | 11.32 (11.27–11.37) |
| 2021 | 10,119 | 36.5 (35.0–37.9) | 11.32 (11.29–11.36) |
| 2022 | 9,881 | 39.5 (38.0–41.1) | 11.15 (11.12–11.19) |
| 2023 | 9,412 | 43.1 (41.8–44.4) | 11.05 (11.02–11.08) |
| 2024 | 9,289 | 40.5 (38.8–42.1) | 11.11 (11.08–11.15) |

*Note. Anemia defined as hemoglobin <11.0 g/dL among children aged 6–35 months.*

## Table S3. Annual distribution of child anemia severity, 2006–2024

| **Year** | **No anemia, %** | **Mild, %** | **Moderate, %** | **Severe, %** |
| --- | --- | --- | --- | --- |
| 2006 | 43.6 | 30.0 | 25.6 | 0.8 |
| 2007 | 45.3 | 27.8 | 26.0 | 0.8 |
| 2008 | 44.8 | 28.2 | 26.1 | 0.9 |
| 2009 | 50.2 | 28.0 | 21.1 | 0.7 |
| 2010 | 50.6 | 26.8 | 21.9 | 0.6 |
| 2011 | 58.6 | 23.5 | 17.3 | 0.6 |
| 2012 | 56.1 | 25.6 | 17.8 | 0.5 |
| 2013 | 53.3 | 28.3 | 17.9 | 0.5 |
| 2014 | 56.4 | 27.0 | 16.1 | 0.5 |
| 2015 | 56.8 | 26.9 | 15.8 | 0.5 |
| 2016 | 56.6 | 27.4 | 15.5 | 0.5 |
| 2017 | 59.1 | 26.2 | 14.4 | 0.3 |
| 2018 | 59.9 | 26.2 | 13.7 | 0.2 |
| 2019 | 62.4 | 25.1 | 12.4 | 0.1 |
| 2020 | 63.2 | 25.7 | 10.9 | 0.2 |
| 2021 | 63.5 | 25.7 | 10.7 | 0.1 |
| 2022 | 60.5 | 26.3 | 13.1 | 0.2 |
| 2023 | 56.9 | 27.9 | 14.9 | 0.3 |
| 2024 | 59.5 | 26.8 | 13.6 | 0.1 |

*Note. Category totals may not sum exactly to 100.0 due to rounding.*

## Table S4. Diagnostics for the main ITS model

| **AR(1) ρ** | **Durbin–Watson (Prais–Winsten residuals)** | **Durbin–Watson (raw residuals)** | **Ljung–Box Q(4), p** |
| --- | --- | --- | --- |
| 0.183 | 1.60 | 1.31 | 0.689 |

*Note. Diagnostics correspond to the final model with Prais–Winsten AR(1) correction.*

## Table S5. ITS with child mean hemoglobin as a continuous outcome

| **Derived estimate** | **Value** | **95% CI** | **p** |
| --- | --- | --- | --- |
| Slope 2006–2010 (g/dL/year) | +0.125 | 0.026 to 0.225 | 0.018 |
| Slope 2011–2016 (g/dL/year) | +0.001 | — | — |
| Level change in 2017 vs CF (g/dL) | -0.507 | -1.169 to 0.156 | 0.123 |
| Post-2017 slope-change β₃ (g/dL/year) | -0.137 | −0.241 to −0.033 | 0.014 |
| Slope 2017–2024 (g/dL/year) | -0.136 | — | — |

*Note. Model with the same temporal specification as the primary analysis: flexible pre-trend, cut-point in 2017, and Prais–Winsten AR(1) correction. CF = counterfactual.*

## Figure S1. Sensitivity analysis: slope change (β₃) using alternative cut-points

*Unadjusted slope-change coefficient (β₃) and 95% CI for models with alternative cut-points in 2016, 2017, 2018, and 2020.*

## Figure S2. Composition-adjusted predicted prevalence

*Annual series of composition-adjusted predicted prevalence among children aged 6–35 months. The vertical dashed line marks 2017.*

## Figure S3. Flow diagram of analytic sample selection

*Figure S3. Flow diagram of analytic sample selection. Sequential selection of the analytic sample from ENDES child records, 2006–2024. The final sample included 123,600 children aged 6–35 months with valid hemoglobin and complete survey design information.*

## Figure S4. Leave-one-out estimates of the post-2017 trend-change coefficient (β₃)

*Figure S4. Leave-one-out estimates of the post-2017 trend-change coefficient (β₃). Points are estimates and bars are 95% confidence intervals; the dashed vertical line is the full primary model estimate.*

## Table S6. Leave-one-out estimates

Rows exclude one ENDES survey year at a time. Coefficients are percentage points per year.

| **Excluded year** | **Excluded n** | **Observed anemia %** | **β₃** | **95% CI** | **p-value** | **Post-2017 slope** | **Delta vs full** |
| --- | --- | --- | --- | --- | --- | --- | --- |
| 2006 | 863 | 56.4 | 3.89 | 1.61 to 6.16 | 0.003 | 0.35 | 0.05 |
| 2007 | 1,731 | 54.7 | 4.04 | 1.71 to 6.38 | 0.002 | 0.35 | 0.21 |
| 2008 | 3,324 | 55.2 | 3.74 | 1.46 to 6.01 | 0.004 | 0.35 | -0.09 |
| 2009 | 3,853 | 49.8 | 4.03 | 1.79 to 6.26 | 0.002 | 0.35 | 0.19 |
| 2010 | 3,902 | 49.4 | 4.05 | 1.86 to 6.24 | 0.002 | 0.35 | 0.22 |
| 2011 | 3,960 | 41.4 | 2.88 | 0.71 to 5.04 | 0.013 | 0.36 | -0.96 |
| 2012 | 4,184 | 43.9 | 3.76 | 1.70 to 5.82 | 0.002 | 0.35 | -0.07 |
| 2013 | 3,861 | 46.7 | 4.14 | 2.01 to 6.27 | 0.001 | 0.36 | 0.31 |
| 2014 | 3,803 | 43.6 | 3.89 | 1.66 to 6.13 | 0.002 | 0.36 | 0.06 |
| 2015 | 10,669 | 43.2 | 3.80 | 1.71 to 5.89 | 0.002 | 0.35 | -0.03 |
| 2016 | 9,452 | 43.4 | 3.85 | 1.68 to 6.01 | 0.002 | 0.35 | 0.01 |
| 2017 | 10,094 | 40.9 | 3.92 | 2.21 to 5.64 | <0.001 | 0.63 | 0.09 |
| 2018 | 9,862 | 40.1 | 3.78 | 1.95 to 5.61 | <0.001 | 0.47 | -0.06 |
| 2019 | 9,695 | 37.6 | 3.68 | 1.70 to 5.66 | 0.001 | 0.25 | -0.15 |
| 2020 | 5,646 | 36.8 | 3.71 | 1.80 to 5.61 | 0.001 | 0.33 | -0.13 |
| 2021 | 10,119 | 36.5 | 3.68 | 2.12 to 5.25 | <0.001 | 0.38 | -0.15 |
| 2022 | 9,881 | 39.5 | 3.60 | 1.88 to 5.32 | <0.001 | 0.34 | -0.23 |
| 2023 | 9,412 | 43.1 | 3.29 | 1.66 to 4.92 | <0.001 | -0.10 | -0.55 |
| 2024 | 9,289 | 40.5 | 4.60 | 1.85 to 7.36 | 0.003 | 0.55 | 0.77 |
